# Case Report: The Coronal Magnetic Resonance Imaging of Three-Dimensional Fast-Field Echo With Water-Selective Excitation Can Identify the Wrapping of Spinal Nerve Fibers Into Subdural Tumors Prior to Operation

**DOI:** 10.3389/fneur.2022.945299

**Published:** 2022-07-14

**Authors:** Tao Tang, Jinghong Yuan, Jianhua Yin, Junchao Zhu, Jingyu Jia, Xigao Cheng

**Affiliations:** ^1^Department of Orthopedics, The Second Affiliated Hospital of Nanchang University, Nanchang, China; ^2^Department of Radiology, The Second Affiliated Hospital of Nanchang University, Nanchang, China; ^3^Institute of Orthopaedics of Jiangxi Province, Nanchang, China; ^4^Institute of Minimally Invasive Orthopaedics of Nanchang University, Nanchang, China

**Keywords:** cMRI, subdural tumors, cauda equina, sensitivity, specificity

## Abstract

**Purpose:**

In the present study, the authors intend to identify the spatial relationship between subdural tumors and spinal nerve fibers of cauda equina prior to operation using the coronal MRI of three-dimensional fast-field echo with water-selective excitation (CMRI).

**Methods:**

In total, 30 case series with surgically and pathologically verified subdural tumors were enrolled in the present study. The spatial relationship between subdural tumors and spinal nerve fibers of the cauda equina was assessed via conventional MRI and CMRI by three experts prior to operation. The spatial relationship between subdural tumors and spinal nerve fibers of the cauda equina was classified using CMRI. The accuracy of imaging observation was determined via intraoperative observation.

**Results:**

Though conventional MRI and gadolinium (Gd)-enhanced MRI (Gd MRI) cannot identify the spatial relationship between subdural tumors and spinal nerve fibers of cauda equina in all cases, CMRI can identify it prior to operation and divide the spatial relationship of spinal nerve fibers of cauda equina with subdural tumors into three types. CMRI shows higher sensitivity (97.44%) and specificity (90.47%) in identifying the spatial relationship of spinal nerve fibers of cauda equina with subdural tumors. Additionally, CMRI also showed a substantial agreement with a kappa value of 0.78.

**Conclusion:**

Herein, the authors first describe a potential novel application that CMRI can successfully identify the spatial relationship between subdural tumors and spinal nerve fibers of cauda equina prior to operation, which play an essential role in making a prudent surgical plan and preventing postoperative nerve damage.

**Summary:**

Intraoperative observation confirms spinal nerve fibers of cauda equina are often wrapped into subdural tumors of the thoracolumbar and lumbar region, which can result in a high rate of sensory and motor dysfunction after the operation due to the unconscious about the wrapping of nerves into subdural tumors prior to operation. To date, there is not an effective strategy to identify the wrapping before operation.

## Introduction

Primary spinal cord tumors (SCNS) represent 2–4% of all central nervous system tumors and are one of the causes of back pain, nerve root pain, and sensorimotor dysfunction in adults and children (1, 2). Subdural tumors located in the dural sac account for almost 30% of SCNS (3). Though conventional MRI and gadolinium (Gd)-enhanced MRI can observe the size and location of subdural tumors and distinguish among intramedullary, intradural extramedullary, and extradural tumors, they cannot be employed to identify the spatial relationship of the nerve fibers of the spinal cord with the subdural tumors. For example, we cannot determine whether the nerve fibers are wrapped into tumors or not. Michael et al. reported that 15 and 5% of the patients developed sensory and motor dysfunction, respectively, after the operation (4). Therefore, it is vital for preventing irreversible nerve damage to identify the spatial relationship of the nerve fibers of the spinal cord with subdural tumors prior to operation. Herein, we first reported that coronal MRI (CMRI) of three-dimensional fast-field echo with water-selective excitation can successfully identify it.

## Materials and Methods

### Case Series

A total of thirty patients with subdural tumors who underwent surgical treatment in our department were enrolled in this study and included 16 men and 14 women with an average age of 53.4 ± 13.06 years old, ranging from 27 to 86 years old. The subdural tumors of 4 cases were located in the cervical segment, 6 cases in the thoracic segment, 7 cases in the thoracolumbar segment, and 13 cases in the lumbar segment. Our pre-study found that it is very difficult in identifying the spatial relationship between subdural tumors and spinal nerve fibers in cervical and thoracic segments using CMRI. Therefore, only the subdural tumors located in thoracolumbar and lumbar segments were involved in subsequent research. Clinical data are shown in Table 1. This study was approved by the medical ethics committee of our hospital and all patients provided their signed informed consent.

**Table 1:** Clinical data and follow-up information.

**Case No**.	**Age (yrs)**	**Sex**	**Tumor location**	**Histology**	**Type**	**MCS**		**VAS**		**ODI**	
						**Preop**	**Postop 1 year**	**Preop**	**Postop 1 year**	**Preop (%)**	**Postop 1 year (%)**
1	27	M	T12	Schwannoma	III	III	I	8	1	66	14
2	63	M	L2	Schwannoma	II	I	I	7	1	44	15
3	58	M	L1	Schwannoma	III	III	I	6	2	54	30
4	57	F	L4	Schwannoma	III	II	I	6	3	56	28
5	60	F	C4	Schwannoma	I	IV	II	7	2	76	16
6	64	M	L3	Schwannoma	III	I	I	5	1	54	12
7	58	F	L5	Schwannoma	III	II	I	7	1	58	22
8	45	F	C6	Schwannoma	I	III	I	7	2	64	10
9	53	M	L3	Schwannoma	I	I	I	7	1	44	8
10	69	M	T12	Ependymoma	I	I	I	6	1	38	10
11	25	F	L1	Schwannoma	III	II	I	5	2	58	12
12	54	M	L2	Meningioma	III	III	I	4	1	66	18
13	68	F	C5	Schwannoma	I	II	I	5	2	63	22
14	86	F	L4	Schwannoma	III	IV	II	3	1	84	30
15	53	F	L1	Lipoma	III	II	I	6	2	45	12
16	60	M	L2	Schwannoma	III	IV	I	5	1	77	17
17	68	F	T12	Schwannoma	III	III	I	7	1	62	15
18	43	M	L2	Schwannoma	III	II	III	5	6	40	56
19	61	M	T4	Meningioma	I	IV	II	4	2	74	12
20	56	F	L3	Meningioma	I	III	I	7	1	64	10
21	56	F	T8	Meningioma	I	III	I	8	1	56	14
22	39	M	T11	Schwannoma	I	II	I	9	2	63	20
23	46	F	T5	Meningioma	I	II	I	5	2	48	10
24	60	M	C7	Schwannoma	I	III	I	4	1	62	22
25	32	F	L3	Teratoma	III	IV	II	7	1	78	18
26	52	M	L2	Schwannoma	I	I	I	6	1	34	8
27	51	F	T11	Schwannoma	I	III	I	8	2	68	14
28	58	M	L1	Meningioma	III	II	I	6	1	56	18
29	48	F	T4	Meningioma	I	II	I	5	1	45	10
30	33	M	L2	Neurofibroma	III	III	I	7	1	69	16

*Type indicates the classification of the spatial relationship between tumor and nerves; MCS, McCormick scores; VAS, Visual Analog Scale/Score; ODI, Oswestry Disability Index*.

### Imaging

Scanning parameters of CMRI refer to our previous research (5). Magnetic resonance nerve root water imaging was performed on the spine of patients with the 3.0T Superconducting MRI scanning system (Skyra; Siemens Healthcare, Erlangen, Germany) with the following scanning parameters: TR 2000 ms, TE 32 ms, FOV 32 cm, turning angle 20°, layer thickness 1 mm, continuous scanning without interval, matrix 352 × 256, with 2 acquisition times. The coronal images were reconstructed by maximum signal intensity projection (MIP) and stereoscopic fluoroscopy (VRT) on the ADW 4.6 software, followed by the 3D rotational observation.

### Classification of the Spatial Relationship Between Tumor and Nerves

The spatial relationship between the tumor and the spinal nerve can be classified into three types. Type I: tumor compresses nerves, the nerves get displaced, but the nerves pass around the tumor and no nerve fibers enter or pass through the tumor. Type II: a part of the nerves enters the tumor and terminates at the end of the tumor. There is no nerve fiber perforation at the distal end of the tumor. Type III: some nerves enter and pass through the tumor.

### Statistics

Statistical analysis was performed using the SPSS version 25 for Windows (SPSS Inc., Chicago, IL, USA). Inter-observer agreement was evaluated by kappa analysis as described elsewhere (6). A kappa value of 0–0.2 was considered a slight agreement, 0.21 to 0.4 as a fair agreement, 0.41 to 0.6 as a moderate agreement, 0.61 to 0.8 as a substantial agreement, 0.81 to <1 as an almost perfect agreement, and 1.00 as the perfect agreement. The chi-squared test was performed to analyze the accuracy of identifying the spatial relationship between the subdural tumor and the nerve on CMRI. An intraoperative observation was considered the gold standard. The sensitivity and specificity were calculated according to the gold standard. *P* < 0.05 was considered to indicate statistical significance.

## Results

**Figure 1:**
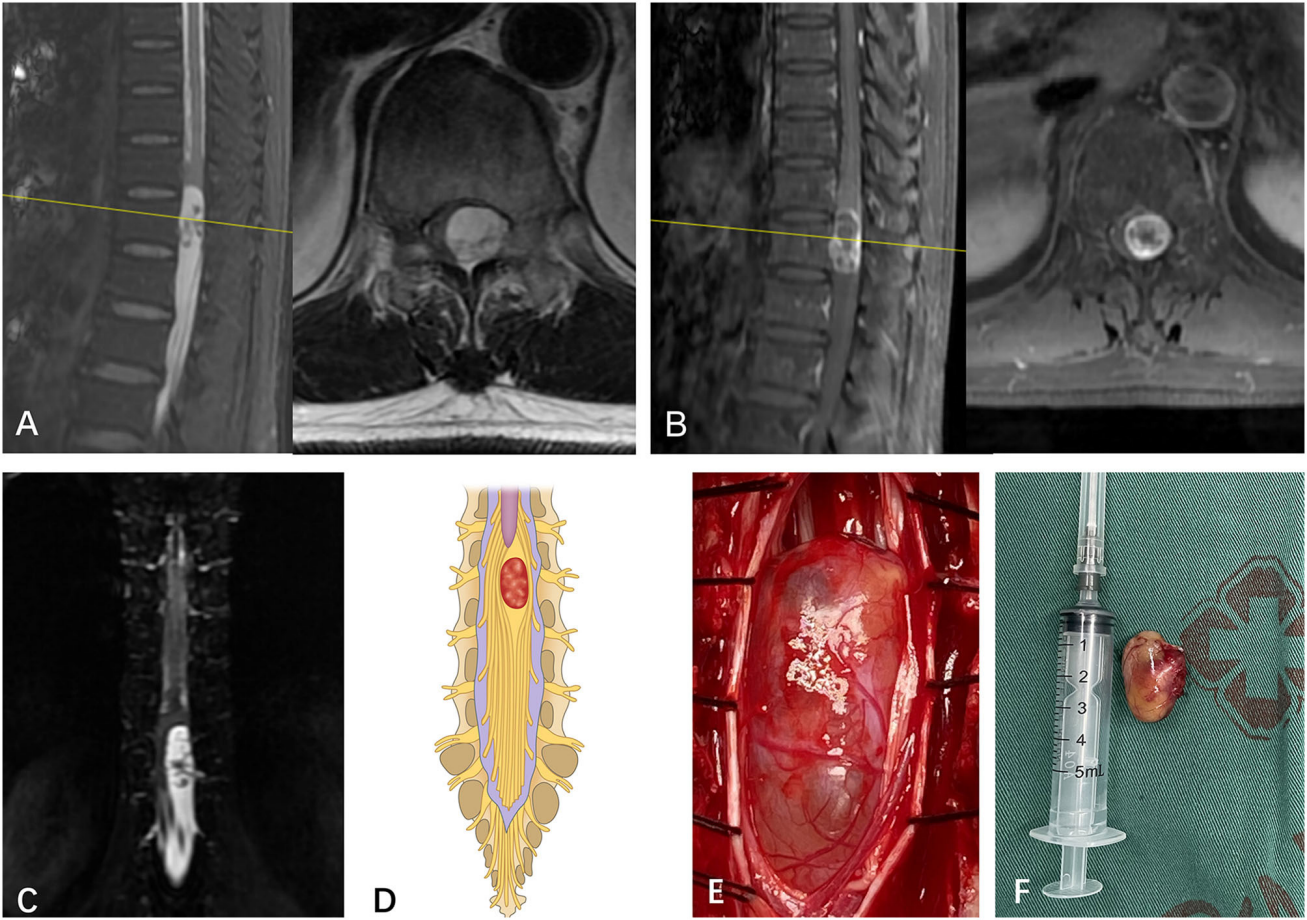
**(A)** A subdural mass with hypersignal T1 and T2 was identified on a segment of L2 in a 55-year-old man via T2-weighted MRI prior to operation. **(B)** The mass presented inhomogeneous enhancement *via* Gd MRI. **(C)** Though both preoperative MRI and Gd MRI did not identify the spatial relationship between the tumor and spinal nerve fibers of cauda equina, the CMRI clearly showed that no nerve fibers enter or pass through the tumor. **(D)** Based on the spatial relationship between the tumor and the spinal nerve, the case was considered type I according to the pattern diagram. **(E)** As expected, an intraoperative observation confirmed that no nerve fibers passed through the tumor (type I). **(F)** The mass was totally resected and confirmed to be schwannoma by postoperative pathology.

**Figure 2:**
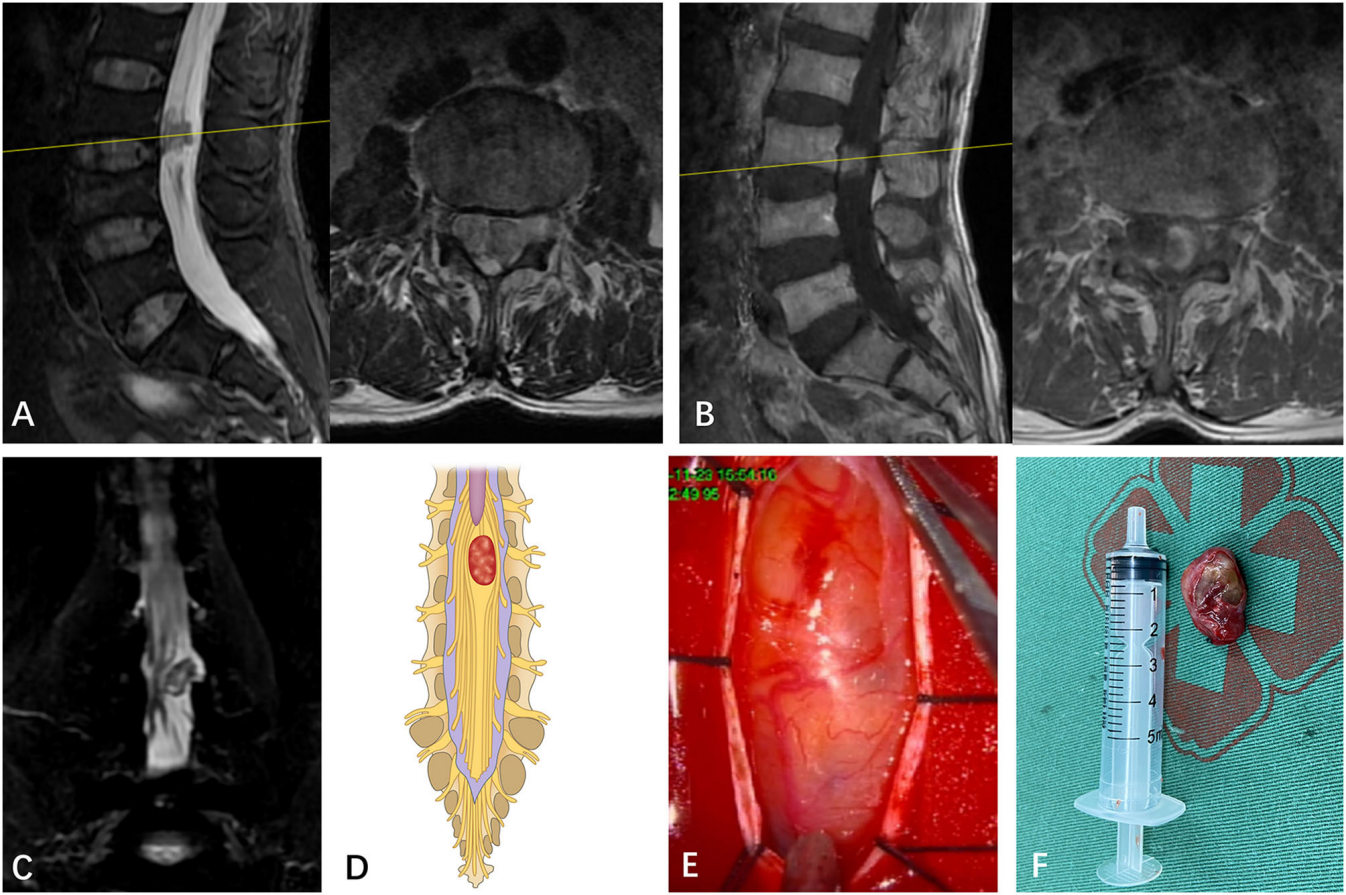
**(A)** A subdural mass with hypersignal T1 and T2 was identified on a segment of T12 in a 58-year-old man via preoperative MRI. **(B)** The mass presented inhomogeneous enhancement via Gd MRI. **(C)** Though both preoperative MRI and Gd MRI did not identify the spatial relationship between the tumor and the spinal nerve, the CMRI clearly showed that a part of the nerves enter the tumor and terminate at the end of the tumor. **(D)** Based on the spatial relationship between the tumor and the spinal nerve, the case was considered type II according to the pattern diagram. **(E)** As expected, an intraoperative observation confirmed that a part of the nerves enters the tumor and terminates at the end of the tumor (type II). **(F)** The mass was resected and confirmed to be schwannoma by postoperative pathology.

**Figure 3:**
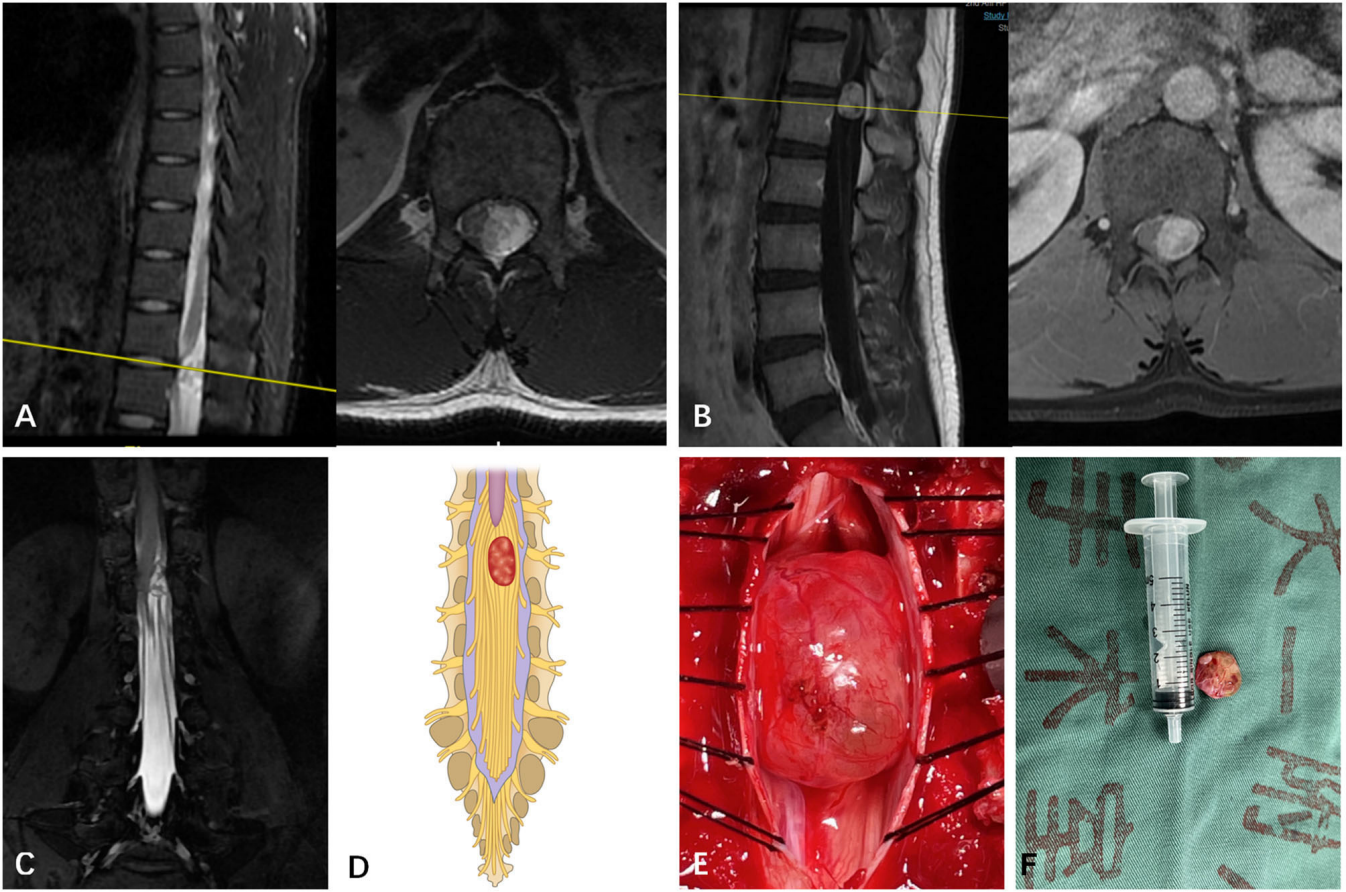
**(A)** A subdural mass with hypersignal T1 and T2 was identified on a segment of T12 in a 27-year-old man via T2-weighted MRI prior to operation. **(B)** The mass presented inhomogeneous enhancement via Gd MRI. **(C)** Though both preoperative MRI and Gd MRI did not identify the spatial relationship between the tumor and the spinal nerve, the CMRI clearly showed that the spinal nerves pass through the tumor. **(D)** Based on the spatial relationship between the tumor and the spinal nerve, the case was considered type III according to the pattern diagram. **(E)** As expected, an intraoperative observation confirmed that nerve fibers passed through the tumor (type III). **(F)** The mass was removed and confirmed to be schwannoma by postoperative pathology.

Clinical data and follow-up information are shown in Table 1. CMRI can clearly demonstrate the spatial relationships between the nerve fibers of the spinal cord and subdural tumors. The sensitivity and specificity of CMRI in ascertaining the relationship between the tumor and the spinal nerve were 97.44 and 90.47%. Additionally, CMRI showed a kappa value of 0.78, which indicated that it was reliable. The spatial relationship between the nerve fibers of the spinal cord and subdural tumors was divided into three types, as shown in the typical case (Figures 1–3). Meanwhile, the limitation of CMRI was displayed in Supplementary Figure S1. The nerve fibers are perfectly displayed in Supplementary Figure S2.

## Discussion

Magnetic resonance nerve root water imaging is a T2 weighted imaging technology that suppresses the fat components between the peripheral and internal nervous tracts and obtains images of only the liquid in the inner membrane of the nervous tract based on heavy T2 weighting combined with fat suppression technology (7). Shen et al. (8) found that, unlike conventional MRI, magnetic resonance nerve root water imaging could clearly depict the full length of the spinal nerve root. Byun et al. (9) observed that the technology could additionally demonstrate the 3D relationship between the nerve root and the intervertebral disc, which is conducive to the discovery of extreme lateral disc herniation. Our previous study confirmed that the sensitivity and specificity of the technique in identifying extreme lateral disc herniation are better than those of conventional MRI (10). Subsequently, it has also been verified by our other study that the CMRI is beneficial in the differential diagnosis of tumor-like disc herniation (5). Considering the advantages of this approach in spinal cord nervous tract imaging, the authors tried to analyze whether it could determine the relationship between subdural tumor and spinal cord nervous tract preoperatively to aid in surgical planning and prognosis prediction.

In the present study, we found that CMRI not only can clearly identify the spatial relationship between the subdural tumor of thoracolumbar and lumbar segments and the spinal cord nervous tract prior to the operation but also can determine that the nerve fibers wrapped into the tumor belong to which nerve root is, as typical case 4. It was extremely helpful for the surgeon to make clear whether the subdural tumor can be totally resected prior to operation *via* CMRI. Moreover, CMRI can assist the surgeon in making decisions on whether the nerve fibers wrapped into a tumor should be resected by weighing the pros and cons. Meanwhile, the surgeon can also inform the patients about the severity of neurological impairment after the operation. Subsequently, we assessed the sensitivity, specificity, and reliability of CMRI in identifying the spatial relationship between the nerve fibers of the spinal cord and subdural tumor. We found that CMRI presented a high sensitivity, specificity, and reliability. This indicated that CMRI holds significance in guiding spine surgeons and neurosurgeons before surgical treatment of subdural tumors.

There are still some limitations to our study. Unlike cauda equina nerve in thoracolumbar and lumbar segments, CMRI cannot identify the spatial relationship of the subdural tumor with nerve fibers of the spinal cord in cervical and thoracic segments. An alternative strategy for the identification is diffusion tensor imaging. However, the reliability of diffusion tensor imaging still needs to be improved due to magnetic susceptibility artifacts at the bone-tissue interface. Only meningioma, neurilemmoma, and teratoma were involved in the study due to the small sample size. A multicenter study ought to be considered using a larger sample size in order to support or refute our findings.

## Data Availability Statement

The raw data supporting the conclusions of this article will be made available by the authors, without undue reservation.

## Ethics Statement

Written informed consent was obtained from the individual(s) for the publication of any potentially identifiable images or data included in this article.

## Author Contributions

TT and JJ contributed to the study concept and design. JYu, JYi, and JZ participated in the data acquisition and analysis. JJ, JYi, and XC evaluated the CMRI. TT, JJ, and XC performed the surgery and perioperative management on the patient. TT and XC wrote the manuscript with contributions from all co-authors. All authors contributed to the article and approved the submitted version.

## Conflict of Interest

The authors declare that the research was conducted in the absence of any commercial or financial relationships that could be construed as a potential conflict of interest.

## Publisher's Note

All claims expressed in this article are solely those of the authors and do not necessarily represent those of their affiliated organizations, or those of the publisher, the editors and the reviewers. Any product that may be evaluated in this article, or claim that may be made by its manufacturer, is not guaranteed or endorsed by the publisher.

## Supplementary Material

Supplemental Figure S1 **(A)** A subdural mass with mixed-signal T1 and T2 was identified on a segment of L1 in a 33-year-old woman via preoperative MRI. **(B)** The lower edge of the mass was enhanced via Gd MRI. **(C,D)** Three-dimensional CT shows partial calcification within the teratomas. **(E)** CMRI cannot identify the spatial relationship between the tumor and the spinal nerve due to calcification.

Supplementary Figure S2 **(A–D)** The continuous coronal images of CMRI can show the full length of the spinal nerve fibers wrapped into the tumor and identify which nerve root is wrapped into the tumor. Therefore, it is a vital tool for spine surgeons and neurosurgeons to make a prudent surgical plan and prevent postoperative nerve damage.

Supplementary Table S1 The type of the spatial relationship between the nerve fibers and the thoracolumbar junction or lumbar subdural tumor was identified via preoperative CMRI and intraoperative observation.
