# Supplementary figures and images for: Case Report: The Coronal Magnetic Resonance Imaging of Three-Dimensional Fast-Field Echo With Water-Selective Excitation Can Identify the Wrapping of Spinal Nerve Fibers Into Subdural Tumors Prior to Operation
Source: Front Neurol. 2022 Jul 14;13:945299. doi: 10.3389/fneur.2022.945299 (PMC9330486; doi:10.3389/fneur.2022.945299)

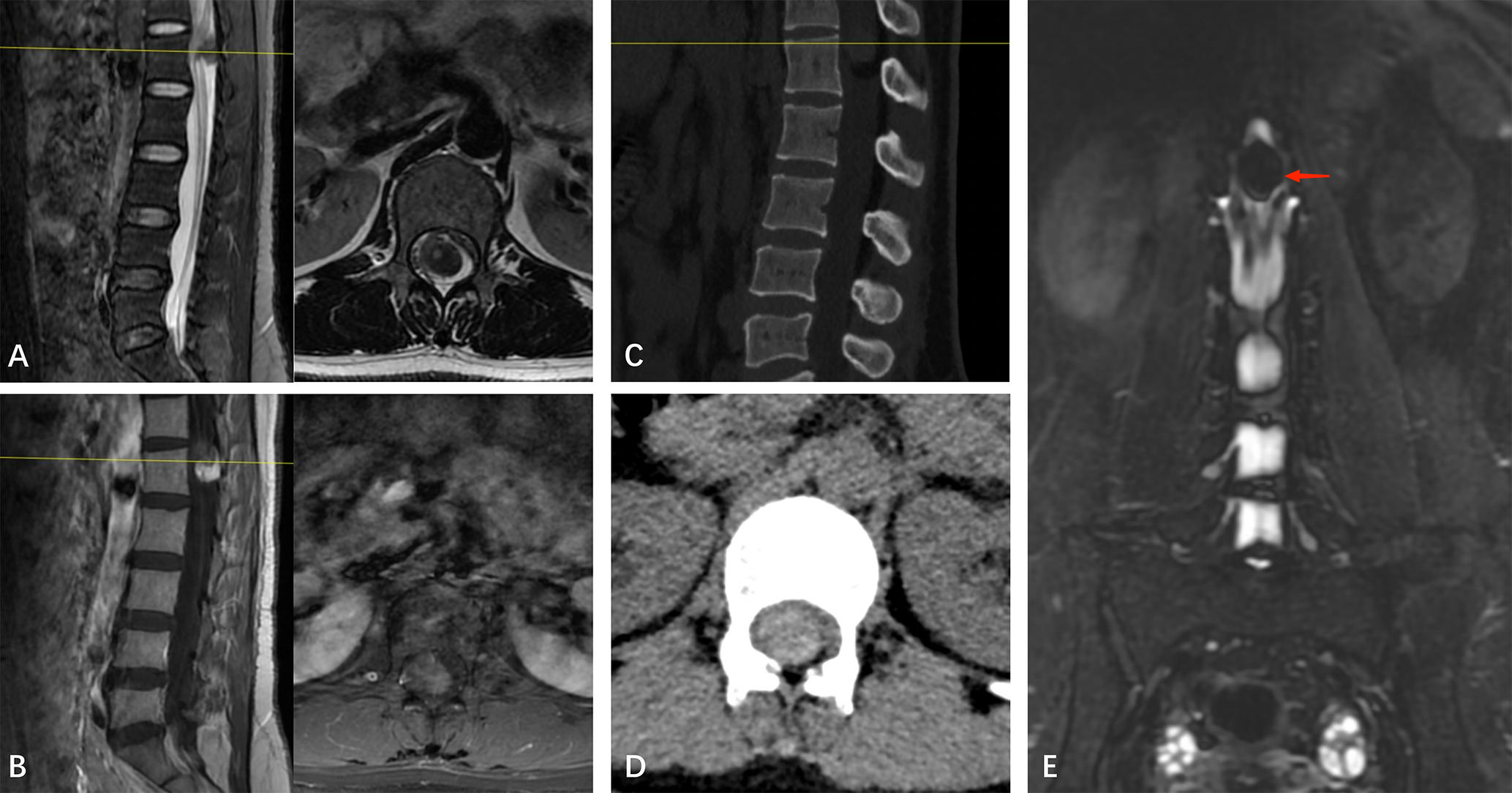

Supplement: Supplemental Figure S1 — (A) A subdural mass with mixed-signal T1 and T2 was identified on a segment of L1 in a 33-year-old woman via preoperative MRI. (B) The lower edge of the mass was enhanced via Gd MRI. (C,D) Three-dimensional CT shows partial calcification within the teratomas. (E) CMRI cannot identify the spatial relationship between the tumor and the spinal nerve due to calcification. [file Image_1.TIF]

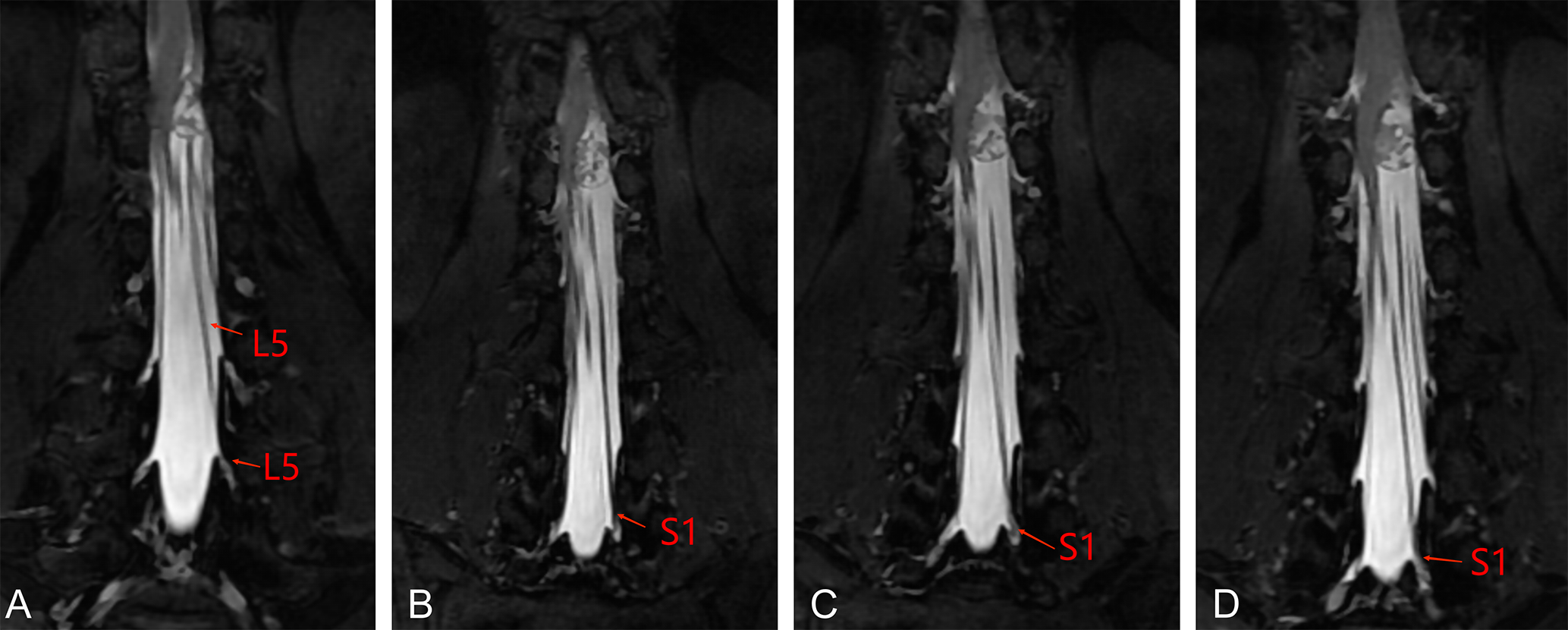

Supplement: Supplementary Figure S2 — (A–D) The continuous coronal images of CMRI can show the full length of the spinal nerve fibers wrapped into the tumor and identify which nerve root is wrapped into the tumor. Therefore, it is a vital tool for spine surgeons and neurosurgeons to make a prudent surgical plan and prevent postoperative nerve damage. [file Image_2.TIF]
