# Supplementary material for: Case Report: The Coronal Magnetic Resonance Imaging of Three-Dimensional Fast-Field Echo With Water-Selective Excitation Can Identify the Wrapping of Spinal Nerve Fibers Into Subdural Tumors Prior to Operation
Source: Front Neurol. 2022 Jul 14;13:945299. doi: 10.3389/fneur.2022.945299 (PMC9330486; doi:10.3389/fneur.2022.945299)
Supplement: Supplementary Table S1 — The type of the spatial relationship between the nerve fibers and the thoracolumbar junction or lumbar subdural tumor was identified via preoperative CMRI and intraoperative observation. [file Table_1.DOCX]

| **Supplemental Table S1. The type of the spatial relationship between the nerve fibers and the thoracolumbar junction or lumbar subdural tumor was identified via preoperative CMRI and intraoperative observation.** | | | | |
| --- | --- | --- | --- | --- |
|  | **CMI** | | | **Intraoperative observation** |
|  | **Jingyu Jia** | **Xigao Cheng** | **Jianhua Yin** |  |
| Type I | 8 | 6 | 6 | 7 |
| Type II | 1 | 1 | 1 | 1 |
| Type III | 11 | 13 | 13 | 12 |

**Kappa value:**

Jingyu Jia and Xigao Cheng: 0.78, p-value <0.001;

Jingyu Jia and Jianhua Yin: 0.78, p-value <0.001;

Jianhua Yin and Xigao Cheng: 1.00, p-value <0.001.
